# The Conditional Analysis of the Agentive Modals: a Reply to Mandelkern et al.

**DOI:** 10.1007/s11406-023-00664-7

**Published:** 2023-06-22

**Authors:** Simon Kittle

**Affiliations:** Department of Philosophy, The School of Philosophy, Religion and History of Science, University of Leeds, LS2 9JT Leeds, UK

**Keywords:** Agentive modals, Modals, Conditional analysis, Abilities, Opportunities

## Abstract

A proper understanding of agentive modals promises to clarify issues to do with free will, know how, and other philosophically interesting topics. In this paper I identify one constraint on, and one structural feature of, trying-based versions of the conditional analysis of the agentive modals. I suggest that the constraint and structural feature together provide a novel account of why the famous Lehrer-Chisholm objection to conditional analyses of ability modals is so powerful. I argue that Mandelkern et al.’s ‘Agentive Modals’ (*Philosophical Review,* *126/3*, 301–343, 2017) conditional analysis of the agentive modals fails to avoid this problem. I also identify two further problems for their account. I close by summarising a number of criteria which any successful semantic analysis of the agentive modals should satisfy.

## Introduction

Agentive modals are expressions which, minimally, express the idea that an agent bears a modal connection to some action-involving event, state or process. In an influential discussion of ‘can’, J. L. Austin identified three classes of agentive modals:

We are tempted to say that ‘He can’ sometimes means just that he has the ability, with nothing said about opportunity, sometimes just that he has the chance, with nothing said about ability, sometimes, however, that he really actually fully can here and now, having both ability and opportunity (Austin, 1956/1979: 230).

According to Austin, ‘can’ may be used to ascribe (i) an ability, (ii) an opportunity to exercise an ability, and (ii) both the ability and opportunity (Austin’s famous “all-in” sense of ‘can’). Many writers have endorsed Austin’s threefold division; typically, the abilities ascribed with Austin’s ability-only meaning of ‘can’ are thought of as intrinsic properties which are *general* in some significant sense, while the abilities ascribed with the “all-in” meaning of ‘can’ are thought of as extrinsic properties which are *particular* or *specific* in some way. Opportunities have received less attention; not infrequently, philosophers follow Austin in holding that it is the possession of an opportunity together with a general ability that gives rise to the possession of a particular ability (Kenny, 1975: 150–2; Horgan, 1977: 407; Berofsky, 2012: 70, 255; Alvarez, 2013: 108; but see Lehrer, 1976: 242 for a cautionary note).

Agentive modals are intrinsically interesting but a comprehensive account of these modals also promises to bring clarity to a number of other areas of philosophy. Two examples are the topics of *free will* and *know how*. Consider the following scenario:

(**Bound Ben**) Ben, an excellent swimmer, has been forcibly bound to a chair. He watches helplessly as a child drowns in a lake (Whittle, 2010: 10).

Those who think free will requires the ability to do otherwise – compatibilists and incompatibilists alike – will suggest that Ben *is not able to* save the child in a sense which is relevant to explaining why Ben is not responsible for failing to save the child. But such theorists will also concede that there is some sense in which Ben *is able to* walk, to swim, and so on, despite being tied to the chair. To explain this, appeal is usually made to the distinction between general abilities on the one hand and particular/specific abilities on the other, both of which can be ascribed using the same agentive modals (see, e.g., van Inwagen, 1983: 13). The distinction between those general ability properties ascribed by the agentive modals and those particular/specific ability properties ascribed by the agentive modals is thus vital to any account of free will which construes it as requiring the ability to do otherwise. Getting clear about these modals (and this distinction) promises to facilitate clearer discussion on the ability to do otherwise requirement on free will.

Or consider the topic of know how. According to Ryle (1949/2009: 14–48), knowing how to do something just is a matter of having an ability to do it. But what kind of ability is required? And what is the nature of that kind of ability? As Maier (2018: 8) has noted, until we are clear about the kinds of property that the agentive modals can ascribe, the precise content of Rylean claim is unclear and for that reason the cogency of critiques of Ryle’s position, such as that offered by Stanley and Williamson (2001), will be inconclusive. In addition to the topics of free will and know how, abilities have been taken as primitives in accounts of knowledge more generally (Greco, 2010: Ch 1), of what it is to possess a substance concept (Millikan, 2000), of intrinsic intentionality (Searle, 1983: Ch 5; 2011), and more. Not for nothing did Austin remark that “in philosophy it is *can* in particular that we seem so often to uncover, just when we had thought some problem settled, grinning residually up at us like the frog at the bottom of the beer mug” (Austin, 1956/1979: 231); this is true even if the ‘can’ in question is restricted to that of the agentive modals.

In this paper I provide an assessment and critique of the conditional analysis of the agentive modals. The conditional analysis was first offered as an account of the sense of ‘can’ relevant to free will (Moore, 1912: Ch 6). Attempts were then made to expand the analysis to cover all ability ascriptions (Nowell-Smith, 1960; Aune, 1963: 398). It was subject to what many took to be devastating critique by Lehrer (1968) and with (among other things) the rise of possible worlds accounts of ‘can’, interest in the conditional analysis fell away. Recently, however, Mandelkern et al. (2017) have argued that a conditional analysis can provide a comprehensive account of the agentive modals. I argue against any revival of the conditional analysis. Section 2 briefly sketches various iterations of the simple conditional analysis of abilities and recounts why it was, by the early 1970s, considered dead. I identify one constraint on and one structural feature of conditional analyses which employ the notion of trying. Together the constraint and structural feature provide a novel account of just why Lehrer’s critique is so problematic for conditional analyses. In Section 3 I argue that Mandelkern et al.’s (2017) recent attempt to resurrect a trying-based conditional analysis fails to avoid Lehrer’s criticism. I also raise two further problems for their account. My assessment of the conditional analyses gives rise to several criteria which I suggest any successful account of the agentive modals should satisfy.

In the remainder of this Introduction I present some representative examples of the range of uses to which the agentive modals are put in ordinary language. These examples feature in the discussion which follows, and I take it that the first constraint on an account of the agentive modals is that it should do justice to the full range of uses without too much awkwardness.

The agentive modals – in English, primarily ‘can’, ‘is able to’ and their cognates – may be used to ascribe general abilities, paradigm examples of which include:

1. Dina is able to drive.
2. Julian is able to walk to the car.

The agentive modals can also be used to ascribe nothing more than an opportunity:

(3) He could have smashed that lob, if he’d been any good at the smash.^1^
(4) Sam can ask her himself; she’s coming round for dinner.

Typical examples ascribing *particular* or *specific* abilities include:

(5) Grigory Sokolov is able to play Chopin’s Prelude in E Minor this evening.^2^
(6) Louise is able to pick Roger up from work at 6pm.^3^

Whittle (2010) pointed out that (at least some) ability ascriptions can be characterised with varying levels of detail, such as in the following:

(7) Dina is able to drive a 16-seater minibus.
(8) Dina is able to drive a 16-seater minibus along narrow, icy roads.
(9) Dina is able to drive a 16-seater minibus along narrow, icy roads one-handed.

In statements (7)-(9) the abilities ascribed to Dina are to be understood as *the-ability-to-drive-a-16-seater-minibus*, *the-ability-to-drive-a-16-seater-minibus-along-narrow-icy-roads*, and *the-ability-to-drive-a-16-seater-minibus-along-narrow-icy-roads-one-handed* respectively, where the hyphenation indicates that the detail is part of the definition of the ability.^4^ Put otherwise, the modal’s prejacent^5^ can be a clause describing an action with an (apparently) arbitrary level of detail.

In addition to the agentive modals above, Mandelkern et al. (2017) have reminded us that ability modals have duals which yield another type of agentive modal. Mandelkern et al. (2017: 1) label these *compulsion modals* and provide the following examples (my numbering):

(10)  Lara cannot but eat another cookie right now.
(11)  I have to sneeze right now.
(12) I cannot *not* climb mountains.

Each of the statements in (1)-(12) ascribe some sort of agentive modality to a single human agent. But the agentive modals may also be used to ascribe an ability or power to (perhaps) any causally potent entity, not just humans qua rational agents, but subsystems of human agents, biological organisms more generally, groups, institutions, artefacts, complex natural systems, inanimate objects, and even ideas or concepts:

(13) The human visual system is able to correctly determine the color of objects in view irrespective of the illuminant.^6^
(14) Hydrolytic proteolytic enzymes are not able to digest fibrous proteins.^7^
(15) Kuhn showed that … [the] leading lights of the scientific community were able to forge ahead by thinking outside the box.^8^
(16) Others will be watching for signs the U.N. is able to adapt to the geopolitical shift away from multilateralism.^9^
(17) It is hoped that the ATLAS detector … at the [Large Hadron Collider] will be able to detect the Higgs boson.^10^
(18) A warmer atmosphere is able to hold more moisture.^11^
(19) That asteroid can cause a mass extinction event on the earth.^12^
(20) [P]hilosophical concepts nurtured in the stillness of a professor’s study can destroy a civilisation.^13^

It might be suggested (I'd like to thank an anonymous reviewer for raising this point) that not all of the above sentences, even those that use the phrase 'is able to', involve the use of agentive modals, and that part of my case against the conditional analysis relies on accruing to the class of agentive modals instances of 'can' and 'is able to' that should be excluded, instances that it would be difficult for any account of the agentive modals to accommodate. However, as will be seen below, Mandelkern et al. (2017: 336-338), whose defence of the conditional analysis is my primary target here, suggest that their account of the agentive modals can handle cases where the grammatical subject is not (obviously) an agent. There are also uses of the agentive modals which are parasitic on one of those uses above, and which appear to describe the abilities or powers that certain hypothetical or idealised agents would have were they actual:

(21) An infant who has reached this babbling stage is able to reproduce the rhythmic pattern of the sentence of the language spoken by the family.^14^
(22) The average person is able to recall approximately 5,000 faces.^15^

In what follows, we will see how this range of uses causes problems for the conditional analysis.

## The Death of the Conditional Analysis

There is a long history of using conditionals to elucidate (sometimes just a subset of the) agentive modals. Hume famously defined being able to act freely in terms conditionals, an idea which was picked up by Moore. Call any account which proposes to analyse an agentive ‘can’ in terms of a single counterfactual a *simple conditional analysis* of ‘can’ (SCA for short). Moore put forward the following version of the SCA:

**SCA-C**

‘S can *phi*’ is true if and only if, if S were to choose to *phi*, S would *phi* (Moore, 1912: 211–2).

Moore offered this as an analysis only of those particular/specific ability ascriptions relevant to free will. After Moore, however, proponents of the SCA sought to develop it into a comprehensive analysis of the general and particular/specific ability modals.

The first choice-point for such thinkers was what to put in the antecedent of the conditional. Moore opted for the verb ‘to choose’ and commentators soon pointed out problems with this: some things can be done without any prior choice, a choice might interfere with the performance of some actions (think highly skilled performances), and sometimes one might choose and fail even when one is able to perform the action in question. Other potential options for the antecedent included ‘to will’, ‘to want’, ‘to wish’, ‘to intend’, and ‘to try’ (Taylor, 1960: 85). Many of these options appeared to be subject to its own set of fatal counterexamples.^16^ Some measure of consensus formed around the idea that the best candidate was ‘to try’. This led thinkers such as Nowell-Smith (1960: 96, 98) and Aune (1963: 398) to offer the following version of the SCA:

**SCA-T**

‘S can *phi*’ is true if and only if, if S were to try to *phi*, S would *phi*.

Why did trying-based analyses appear to be more successful? To answer this it is helpful to consider how the SCA works. The SCA is what I will call a *compound* analysis of the ability modals: it works by breaking down the action into two elements (hence ‘compound’) which are then (re-)connected by a counterfactual. There appear to be two ways of doing this. The first way identifies some entity which is either an element in the action’s causal history or part of the action itself. Call any account taking this first way a *componential compound account*. This is the route taken if we fill the antecedent with a verb such as *choosing*, *willing*, *wanting*, and so on. In the SCA, terms such as ‘choose’, ‘will’, ‘want’ are interpreted as mental events which precede (or are part of) the action. But there is a problem: if one takes this componential compound route, then in picking one of these terms to use in the antecedent, one is thereby importing a set of substantive action-theoretic assumptions into the analysis of the ability modals. For example, if one employs ‘to intend’ in the antecedent, one is thereby committed to the view that all action is accompanied by an intention. As such, counterexamples to the thesis that all action is accompanied by an intention will generate counterexamples to one’s proposed analysis of the ability modals.

The second way of pursuing a compound analysis is to employ ‘to try’ in the antecedent. Most trying-based compound analyses are distinctive in that they employ a concept of trying which does not construe trying as an *element in* the action’s causal history or as a *part of* the action but which instead *equates trying with acting*. O’Shaughnessy’s account of trying is representative of the notion of trying employed:

Trying consists in doing, intentionally and with just that purpose, whatever one takes to be needed if, the rest of the world suitably cooperating, one is to perform the action (O’Shaughnessy, 1973: 369).

With any such trying-based compound analysis, it is *not* the case that *trying* is conceived of as a distinctive species of action, in the way that *choosing* (for instance) might be. Rather, the action cast in terms of trying is simply *another description of the action performed*, a description that doesn’t entail the successful performance of the action which occurs in the modal’s prejacent. Trying-based analyses count as *compound analyses* in my terminology because they “break up” the action into *two descriptions* which are connected by the counterfactual. But because trying-based accounts *identify* trying with acting – not with a particular type of action, but with acting itself – they import fewer action-theoretic assumptions into the analysis of the agentive modals: typically, they are committed to the *existence of* intentional action (when an account of trying such as O’Shaughnessy’s is used), but they are not drawn on *the nature of* intentional action. This makes trying-based analyses much more flexible than componential compound analyses.

Still, some counterexamples to trying-based analyses arose. Chisholm (1964: 23), for instance, pointed to actions which people 1992*can* do but *cannot try to* do. Chisholm thought that *instrumentally basic actions* – actions one does directly, and not by doing anything else – such as *closing one’s eyes* fell into this category. This type of counterexample relies on the controversial claim that basic actions cannot be tried. Another type of counterexample are those cases of skilful action “where trying too hard seems to hinder performance” (Montero, 2016: 147). The idea here is that, if a seasoned tennis pro (say) *pays too much attention* to the stroke at hand, or *tries too hard* to play well, the tennis player will lose that automatic flow sometimes needed for success. (Below I highlight another class of counterexamples which concern non-minded agents who cannot try).

I submit that these sorts of counterexamples – to both types of compound analysis (non-trying-based componential analyses and trying-based analyses) – arise from the same source: the importing of substantive action-theoretic assumptions into the analysis of the agentive modals. Trying-based analyses import fewer such assumptions; but they still import some. This is a problem because there are multiple concepts of action, one or more of which the modals become unable to express if substantive action-theoretic assumptions are incorporated into their analysis. For example, alongside the notion of “full-blooded” human agency on which philosophers tend to focus (Velleman, ), there are purposive actions which are not intentional (Frankfurt, 1978), arational actions (Hursthouse, 1991), unintentional actions, and perhaps actions which are neither intentional nor unintentional (Lowe, 1978). There are also things people do, such as idly drumming one’s fingers on the table, which are (at least) cases of agentive activity (Frankfurt, 1977). Moreover, there is a permissive concept of action according to which to act just is to cause some change (see, e.g., Brown, 1968: 35). This latter notion of action was more prevalent in philosophy before theories of event causation came to dominate, but examples (17) and (19) suggest that the agentive modals can still be used to attribute a power to act in this sense:

- (17) It is hoped that the ATLAS detector … at the [Large Hadron Collider] will be able to detect the Higgs boson.
- (19) That asteroid can cause a mass extinction event on the earth.

Chisholm (1964: 22) suggested that the first step in repairing **SCA-T** would be to modify it like so:

**SCA-T2**

‘S can *phi*’ is true if and only if, if S were to try to *psi*, S would *phi*.

In **SCA-T2**, the action which the agent tries to perform is not that to which the ability modal pertains. And precisely because the counterfactual connecting the agent’s *psi*-ing and the agent’s *phi*-ing is such a weak connection (e.g., the agent need not have any awareness they are *phi*-ing), this produces a very flexible analysis: the prejacent of the agentive modal can now scope over unknown, unintended, and/or causally distant results of the agent’s trying.

Still, both Chisholm (1966) and Lehrer (1968) pointed out that **SCA-T2** faces another problem, namely, that the truth of the conditional in the analysans is not sufficient for the truth of the analysandum. Lehrer’s counterexample to **SCA-T2** became the *locus classicus*:

**Red Candy**

Suppose Larry is offered a bowl of red candy. Larry does not try to take one of the red sweets because he has a pathological aversion to such candy. However, it is logically consistent to suppose that (i) if Larry had tried to take a red candy, Larry would have taken one, and that (ii) given Larry’s pathology, Larry is unable to try to take one.

(Adapted from Lehrer, 1968: 32).

The problem here is that, because the conditional (clause (i) of the passage cited) is true, **SCA-T2** predicts that Larry is able to take a red candy. But it is clearly consistent to hold that Larry is unable to *try to* take a red candy, and on the assumption that Larry can only take a candy by trying to take a candy (and given the nature of the example we can simply stipulate this to be the case), it seems to follow that Larry is unable to *take* a red candy. Call this the Chisholm-Lehrer objection (Chisholm’s objection was framed slightly differently, but the details are not relevant here).

In response to this problem, Chisholm proposed what I will label a *hybrid conditional analysis* – an analysis which required (alongside the truth of the conditional) an extra condition to be satisfied. Chisholm’s 1967 account, which is framed as an analysis of *having something within one’s power* (as opposed to an analysis of ‘can’/‘is able to’), is as follows:

**D1**

[Someone’s] making *A* happen at *t’* is directly in his power at *t*, provided only: (a) there is an event *X* such that his undertaking at *t* to make *X* happen would cause *A* to happen at *t’*; and (b) there is no sufficient causal condition at *t* for his not undertaking at *t* to make *X* happen (Chisholm, 1967: 417).

In fact, this is only part of Chisholm’s account; his full account is complex and includes definitions of both *having something* ***directly*** *in one’s power* and of *having something* ***indirectly*** *in one’s power*. However, that complexity is not pertinent to the present point so I ignore it here. In **D1**, clause (a) is roughly equivalent to **SCA-T2**. Someone can – or, has it within their power to – make *A* happen only if, were the agent to undertake (i.e. to try) to make *X* happen, the agent’s *X*-ing would cause *A* to happen.

Clause (b) contains Chisholm’s response to the Chisholm-Lehrer problem. It consists of an extra, non-conditional criterion on the truth of ‘S can make *A* happen’, namely, that it must be causally possible that the agent undertakes (i.e. tries) to make *X* happen. Notice what is going on here: on the trying-based SCA, being able to X is analysed in terms of doing X if one Y-s. Now, to avoid the **Red Candy**-type examples, the theorist must include some details on whether and in what sense *the agent can Y*. However, since Y-ing is trying, and since trying has been equated with acting per se (and not just equated with a particular species of action), giving an account of whether and in what sense the agent can Y *just is* the original problem of analysing the agentive modals (I return to this point below). It is not surprising, then, that Chisholm’s proposed “extra condition” is a modal criterion on the agent’s Y-ing. The modal criterion Chisholm puts forward in (b) is, in effect, Chisholm’s analysis of what it is to be able to do something. Unfortunately, but unsurprisingly, Chisholm’s suggested criterion – that the agent’s Y-ing be *causally possible* – is far too weak. To see this consider the following case:

Suppose that the universe is indeterministic and this indeterminism affects human cognition. Suppose too that it is 1pm on Saturday afternoon and Kasumi is deliberating about what to do; she’s thinking she’ll either go on a bike ride or read a novel. Unbeknownst to Kasumi, there is a 0.01% objective probability that Kasumi will receive a phone call at 1:05pm informing her that she’s just won a new car (due to being entered into a prize draw in virtue of having made a certain purchase last month, although she was entirely unaware of this fact). If she were to receive such a phone call, all she would need to do to secure the prize would be to confirm her personal details and give the details of the item purchased. As things happen, Kasumi does not receive the phone call.

Given that scenario, consider the action of *confirming she’d like the prize at 1:06pm*. It should be evident that as far as this action goes, Kasumi satisfies clause (a) of Chisholm’s **D1**: there is an event *X* (Kasumi’s giving her personal details and details of the item purchased) such that Kasumi’s undertaking at 1:06pm to make *X* happen would cause *A* (her securing the prize) to happen at 1:07pm. That is, *were* Kasumi to try to confirm her details and the details of the item purchased, she would secure the prize. That conditional is true of Kasumi even though, as things unfold, she never becomes aware of even having entered the prize draw. But it’s also true that Kasumi satisfies clause (b) of Chisholm’s **D1**. Since there is an objective probability that Kasumi will receive the phone call at 1:05pm, there are no causal conditions prior to 1:05pm which are sufficient for Kasumi’s not confirming she’d like the prize. Thus, according to **D1**, it is directly within Kasumi’s power to confirm she’d like the prize. But this is false. It is *possible that* Kasumi confirms she’d like the prize at 1:06pm, but it is not *possible for* Kasumi to confirm this: it is not (prior to 1:05pm) within Kasumi’s power to confirm she’d like the prize at 1:06pm (see Shabo, 2014 and Kittle, 2017  for further discussion of examples of this type).

The lesson to draw from the above is that the Chisholm-Lehrer objection reveals trying-based versions of the SCA to be viciously circular, something which is not necessarily true of other versions of the SCA. Consider a version of **Red Candy** which applies to **SCA-C**: to be able to take a red candy, the agent must choose to do so, but the agent is pathologically averse to so choosing. A proponent of **SCA-C** could respond to such a counterexample like so: choosing is acting, but a distinctive type of acting, so in view of this counterexample, I now offer **SCA-C**, not as an entirely general analysis of ability modals, but as an analysis of what it is to be able to perform all types of action except choosing, I augment **SCA-C** by saying that the agent must be able to choose, and I accept that a different analysis of what it is to be able to choose must be provided. This would be a cost to the account, but it shows that the Chisholm-Lehrer objection is not fatal to choice-based versions of the SCA.

By contrast, no such response is available to the proponent of a trying-based version of the SCA. It is a structural feature of all plausible trying-based versions of the SCA that they equate trying with acting per se. They are constrained in this way so as to avoid encountering specific counterexamples faced by componential analyses, but in satisfying this constraint, the Chisholm-Lehrer objection becomes far more problematic. For the proponent of a trying-based version of the SCA, the question, ‘But can the agent try to *phi*?’, is not just a question about a distinctive type of ability and action, or a question which might show the proposed analysis to be less than fully general, or in some way incomplete; rather, the question ‘But can the agent try to *phi*?’ just is the original question. The Chisholm-Lehrer objection thus reveals trying-based versions of the SCA to be viciously circular.

Few philosophers in the 1960s and 70s attempted to patch up the conditional analysis. One suspects two factors led to the loss of interest in conditional-based analyses of the agentive modals. First, the development of possible worlds semantics promised a new way forward without the need to invoke conditionals. Lewis (1976) sketched a semantic analysis of ‘can’ in such a framework using the existential quantifier, and Kratzer (1977,1981,1976; Horgan, 1979; Lehrer, 1990; Campbell, 1997) developed the idea in depth. Meanwhile, philosophers primarily interested in free will presented analyses of the ‘can’ of free will directly in terms of possible worlds (e.g. Lehrer, ). Second, Frankfurt’s (1969) attack on what is usually called the Principle of Alternative Possibilities^17^ convinced many that free will does not require the ability to do otherwise, and for many compatibilists this undercut the motivation of seeking a conditional analysis of ‘can’.

## The Resurrection of the Conditional Analysis?

In their recent article ‘Agentive Modals’, Mandelkern et al. (2017) attempt to resurrect the conditional analysis and suggest that it yields a plausible analysis of particular/specific ability modals, general ability modals and the compulsion modals. After explaining their account and noting its similarities to Chisholm’s account, I argue that it faces three problems. First, it fails as an analysis of particular/specific ability modals for the same reason Chisholm’s analysis failed. Second, *pace* Mandelkern et al., general ability modals are not always assessed against a set of “normal conditions”. And third, the extension of the account to cover agents which cannot try is unsuccessful.

### Mandelkern et al.’s Analysis

Mandelkern et al.’s analysis of specific abilities is as follows (Mandelkern et al., 2017: 314):

**Act Conditional Analysis**

⟦ S can *phi* ⟧^c, w^ = 1 iff ∃A ∈ *A*_S, c, w_ : ⟦ *phi*(S) ⟧^c, f^_c_^(S tries to *A*, w)^ = 1.

In this schema, *S* is the agent, *phi* is the action, *c* is the context, and *w* is the world. *A* is a set of actions which are “practically available” to *S* in context *c* at *w*, and *A* is one member of that set. *f*_*c*_ is the selection function borrowed from Stalnaker’s theory of conditionals: it is a “contextually supplied function” from the proposition ‘S tries to *A*’ and world w to the closest world where ‘S tries to *A*’ is true (Mandelkern et al., 2017: 308). Informally, this analysis says that an utterance of ‘S can *phi*’ is true just in case S is in a situation where there is an action, *A*, which is practically available to S, and were S to try to *A*, S would *phi*. Put directly in terms of possible worlds: ‘S can *phi*’ is true just in case S is in a situation where there is a practically available action, *A*, and in the closest world where S tries to *A*, S *phi*-s.

Mandelkern et al. define *practical availability* like so:

we suggest that an action *psi* will typically count as practically available for S in a context *c* just in case S could reasonably conclude in favor of doing *psi* with respect to the goal of doing the prejacent or its complement (Mandelkern et al., 2017: 319).

Mandelkern et al. say that the “key difference” between their account and previous presentations of the SCA is the suggestion that what the agent tries to do might be different from the action performed. As we’ve already seen, however, this is mistaken. By the mid-1960s Austin and Chisholm had both identified the problems which motivate the move from **SCA-T** (‘if S were to try to *phi*, S would *phi*’) to **SCA-T2** (‘if S were to try to *psi*, S would *phi*’). Mandelkern et al. note that Chisholm (Chisholm, 1964: 24) presented such a version of the SCA, but also note that he did not endorse it; they speculate that this was because Chisholm thought it untenable due to the Chisholm-Lehrer objection. What Mandelkern et al. don’t mention is that in later work Chisholm (1967) presented and endorsed a version of the SCA which he took to address the Chisholm-Lehrer objection. This was Chisholm’s **D1** analysis discussed above. I now lay out three problems for the Mandelkern et al. account. In addition to the problems outlined below, conditional analyses of abilities are known to suffer from several other structural problems (e.g. those arising in connection with finks, the gradability of abilities, the comparative nature of abilities, etc.); my focus here is on those problems which Mandelkern et al. specifically set out to address.

### Problem 1: The Chisholm-Lehrer Objection

Mandelkern et al. include the practical availability requirement (and so develop a hybrid conditional analysis) in an attempt to avoid counterexamples to the SCA, including the Chisholm-Lehrer objection (Mandelkern et al., 2017: 318). They explain how they envisage the notion solving Lehrer’s **Red Candy** example as follows:

If Larry is so phobic that we cannot even entertain the possibility of his trying to take the candy, then we may well not treat taking the candy as practically available for Larry. In that case, we will predict that ‘Larry can take the candy’ is false, even if ‘If Larry tried to take the candy, he would’ is true (Mandelkern et al., 2017: 317).

The basic idea is the same as with Chisholm’s clause (b): we add some extra condition to the analysis which agents such as Larry, who *cannot* try, fail to satisfy. In Mandelkern et al.’s case, the extra condition is that there must be some action which is “practically available” to the agent which if performed would reach the intended goal. As the passage just quoted makes clear, Mandelkern et al. think it plausible that Larry fails to satisfy this condition because he may be so phobic that he cannot even entertain the thought of trying to take the candy. If so, Larry would not be able to “reasonably conclude in favour of taking the red candy”, and Mandelkern et al.’s analysis would give the right result.

This attempt at solving the problem is no more successful than Chisholm’s. True, we *can* envisage Larry being so phobic of candy that he cannot even “conclude in favor” of trying to take the candy. But we *need not*. After all, it is psychologically possible for an agent to have a phobia without realising it. As such, we can simply stipulate that Larry does not know he has a phobia and that while Larry is phobic enough to mean he *cannot try* to take the candy he is not so phobic that he cannot deliberate or “conclude in favor” of it. Of this expanded scenario, Mandelkern et al.’s analysis predicts that Larry can take the red candy, which is incorrect.

I have argued that the truth of the conditional in Mandelkern et al.’s analysis together with the satisfaction of practical availability is not sufficient for the truth of ‘S can *psi*’. It’s also doubtful that an action’s being practically available is necessary for an analysis of ‘S can try to *psi*’. First, there are things people do which cannot be reasonably concluded in favour of given the agent’s goals. Many irrational actions fit this bill. Second, in statements such as (17) (‘It is hoped that the ATLAS detector … at the [Large Hadron Collider] will be able to detect the Higgs boson’) and (19) (‘That asteroid can cause a mass extinction event on the earth.’) the subject of the agentive modal cannot conclude in favour of anything because the subject has no mind (this problem is discussed further below). Mandelkern et al. might reply by stressing their claim that the notion of practical availability is context sensitive, such that it may need suitable reinterpretation in some contexts. But if this reply is to work, we need some account of the notion of context sensitivity in play, and how it might apply in the cases above. Any account which doesn’t offer this is (at best) incomplete. And to the degree that it is doubtful that any notion of practical availability/concluding in favour of will apply to, e.g., warmer atmospheres (example 18) and asteroids (19), it is doubtful that Mandelkern et al.’s account will succeed as a comprehensive analysis of all particular/specific ability modals.

I stated in Section 2 that the Chisholm-Lehrer objection shows trying-based versions of the SCA to be viciously circular because such analyses shift the focus to the question of whether and in what sense the agent can try which just is, for proponents of trying-based analyses, the original question (due to the equating of trying with acting). This is why the “extra conditions” in the analyses of Chisholm and Mandelkern et al. incorporate modal claims (recall that Chisholm’s **D1** clause (b) requires that it be *causally possible* that the agent undertake to make *X* happen; Mandelkern et al.’s notion of practical availability requires that the agent *could* conclude in favour of the action). Each “extra condition” is – because *trying* has been *equated* with acting – an analysis of the modals we are attempting to analyse. The “extra condition” is where the action is.

It might be suggested that this is less of a problem for Mandelkern et al.’s account because they explicitly deny that they are offering a reductive account (Mandelkern et al., 2017: 323). But this thought does nothing to alleviate the problem caused by the Chisholm-Lehrer objection, which could be stated as a dilemma: either the “extra condition” will be an inadequate account of what it is to be able to try, in which case the analysis will fail to avoid **Red Candy**-style counterexamples; or the “extra condition” will be an adequate analysis of what it is to be able to try, in which case, that analysis of being able to try should just be offered as a general analysis of what it is to be able to do something (since on this view trying just is acting), whereupon the conditional aspect of the analysis becomes otiose. (In the sections that follow, we will see that Mandelkern et al.’s stipulation that their notions of *practical availability* and *concluding in favour of* are context sensitive and may even apply to non-minded agents casts doubt on the idea that their analysis might elucidate the agentive modals without being a reduction thereof.)

### Problem 2: General Ability Modals are Not Always Assessed Against a Set of “normal conditions”

Mandelkern et al. use their Act Conditional Analysis of particular/specific ability modals to develop an account of general ability modals. Their analysis is sophisticated, but many of the fine details need not concern us here. The problem I wish to raise for their account is that it analyses general ability ascriptions using a generic operator (which they label GEN) which has “roughly the meaning of ‘generally’” (Mandelkern et al., 2017: 329) and which ties the truth conditions of general ability modals to a set of contextually determined normal conditions. As they explain (here, ‘s’ ranges over situations, and ‘J’ refers to the agent):

Informally, a generic ability ascription with the surface form [J can *phi*] says of J that, in enough normal situations *s*, there is a practically available action at the time of *s* that is such that if J tries to do that action, J brings it about that [*phi*]^c^ holds of her at that time (Mandelkern et al., 2017: 330).

Mandelkern et al. add that, “To make these truth-conditions plausible, we must assume that what counts as a contextually normal situation depends on the prejacent” (Mandelkern et al., 2017: 330). For example, consider:

- (23) George can make great ratatouille.

What counts as normal conditions for (23) is determined in part by ‘makes great ratatouille’ and will therefore include “only those [situations] where George has all the ingredients, tools, and so forth, needed to make ratatouille” (Mandelkern et al., 2017: 330).

Unfortunately, such a scheme will not be able to give the right results for all general ability modals because (i) sometimes the modal’s prejacent and the context do not determine any set of normal conditions, and (ii) sometimes we ascribe general abilities which pertain to non-normal conditions. To see the first point, consider the following example:

(**Mary and Marty**) Mary can memorise a shuffled pack of cards, but only if she’s listening to heavy metal music (and nothing else), which she uses to create a ‘memory palace’ to aid her; Marty can memorise a shuffled pack of cards, but only if he’s listening to classical music (and nothing else), which he uses to create a ‘memory palace’ to aid him. Neither can perform the feat of memorisation in silence, nor while listening to multiple kinds of music at once (Kittle, 2022: 1299).

In this example, Mary and Marty are each able to memorise a pack of cards, yet there is no single set of conditions in which both can memorise. Mary’s ability “pertains to” those scenarios where there is only heavy metal music playing; Marty’s ability “pertains to” those scenarios where there is only classical music playing. And the conditions are not additive: in a scenario where there is heavy metal music playing *and* classical music playing, neither Mary nor Marty can memorise (we can simply stipulate that (say) it’s too hard to concentrate in such scenarios).

We can, however, easily imagine a context where the following sentence is true and attributes a memorisation ability to Mary:

- (24) Mary can memorise a shuffled pack of cards.

Similarly, we can imagine a context where the following is true and attributes a memorisation ability to Marty:

- (25) Marty can memorise a shuffled pack of cards.

In sentences (24) and (25) one and the same prejacent is used to attribute abilities to Mary and Marty. But by hypothesis Mary and Marty’s have different memorisation abilities. What then should we say about this sort of case? Is there a common ability they both possess which these sentences ascribe? Or does the prejacent together with some further feature of context determine the ability ascribed?

There are two ways that a Mandelkern et al.-style account might construe a case like this. First, it might be suggested that the prejacent determines a disjunctive set of circumstances – memorising with heavy metal music playing *or* memorising with classical music playing – such that the two sentences ascribe two tokens of the same type-ability. This set of disjunctive circumstances would, in effect, be the normal conditions for the prejacent ‘memorising a shuffled pack of cards’. This would enable the Mandelkern et al. account to explain the truth of (24) and (25) in the imagined contexts above. I agree that we *can* ascribe such a gerrymandered ability as the-ability-to-memorise-a-shuffled-pack-of-cards-either-with-heavy-music-playing-or-classical-music-playing-but-not-both, but I deny that this avoids the objection. There are two problems. The first is that there might be any number of conditions which people use (and need) to perform the feat of memorising a shuffled pack of cards: some might need soul music, others might need to have their eyes closed, some might need utter silence, and so on. We can imagine contexts where ‘can memorise a shuffled pack of cards’ is said truly of people possessing an ability of any of the aforementioned variations, and so this approach would mandate including all such sets of circumstances. It is implausible that this is what’s going on. The second problem with this approach – a problem which appears fatal – is that even though we could, if we really wanted to, attribute such a disjunctive ability, we can also attribute an ability which pertains to just a single set of circumstances. Suppose, for example, some friends of Mary and Marty are at a heavy metal gig and are discussing whether anyone could exhibit the party trick of memorising a pack of cards. In this context, an utterance of (24) would be true while an utterance of (25) would be false. But if the prejacent ‘memorise a shuffled pack of cards’ determined a disjunctive set of circumstances, with one disjunct for each set of circumstances in which someone could memorise a pack of cards, then both (24) and (25) would be true. So this first approach is unsuccessful.

Now, it might be complained that all I have offered here is a standard context shifting argument and that the Mandelkern et al. account has the resources to deal with such cases. After all, although Mandelkern et al. say that what counts as normal conditions *depends on* the prejacent, they do not say that the prejacent (alone) *determines* the set of normal conditions. Thus, normal conditions might also depend on other features of the context of utterance.

This brings us to the second way that a Mandelkern et al.-style account might address such cases: an appeal to the wider context. But what features of the wider context might contribute (together with the prejacent) to determining what counts as normal conditions? The gerrymandered nature of the Mary and Marty case casts doubt on the idea that there is any notion of normal conditions for ‘memorising a shuffled pack of cards’ (context dependent or otherwise). It’s entirely opaque what should count as normal conditions for a prejacent such as ‘memorising a shuffled pack of cards’. There *may be* settled community norms in given contexts for what counts as normal conditions for, say, *walking*, but there are none for an action such as *memorising a shuffled pack of cards*. The following suggestion may be made: given that the friends *are currently at* a heavy metal gig, *being at a heavy metal gig* counts as normal. But this won’t work: just imagine the group of friends at the heavy metal gig discussing who will be able to memorise a shuffled pack of cards tomorrow, when the group will be at a classical music concert.

Perhaps it will be suggested that Mary or Marty themselves enter into the context. That is, merely by attributing the ability now to Mary, and now to Marty, the context has shifted, with Mary and Marty forming part of the respective contexts. If Mary and Marty can form part of the contexts, then what count as normal worlds for Mary need not count as normal for Marty, so the account could, after all, explain the difference above. But this won’t work either. Imagine that Mary can memorise a shuffled pack of cards to heavy metal music (as above) and that she can also memorise a shuffled pack of cards to soul music but that these are different abilities: different parts of Mary’s brain are involved in the different memorisation tasks, and the memorisation process Mary uses in each case is different. In such a case, if a speaker wanted to attribute to Mary the ability-to-memorise-to-soul-music and not the ability-to-memorise-to-heavy-metal-music including Mary in the context won’t help isolate what needs to count as ‘normal conditions’.

To handle such a case, the proponent of a normal conditions-based approach might claim that the environment *of the utterance* which ascribes the ability generates and is included in the context. This reply might provide a solution – but only if it is allowed that *the speaker’s intentions* are part of the context. But in that case, it is an appeal to what the *speaker intends to mean* that does the work, not any appeal to some notion of *normal conditions*. We are simply labelling whichever circumstances yield the truth of the speaker’s utterance as ‘normal’. The idea of ‘normal conditions’ has now become vacuous.

What the example shows is that we need to allow the relevant assessment circumstances to vary arbitrarily. There *may* be some cases where general ability modals are assessed against some background of widely agreed upon normal conditions. But this is not always (and perhaps not typically) true of general ability modals. So we can affirm that the relevant circumstances are determined by the context and the prejacent, but only if we allow the speaker’s intentions to be part of the context. This is the solution I favour. But appealing to the speaker’s intentions would, I take it, be contrary to the spirit, if not the letter, of Mandelkern et al.’s account.

The point just made applies, not only to gerrymandered ability modals such as ‘is able to memorise a pack of shuffled cards’, but also to modals such as ‘is able to walk’. Consider the following scenario:

Ann plans to take her elderly father, Julian, out for coffee and, not having visited in a while, she says to her brother, ‘Is dad able to walk to the car?’ Both Ann and her brother know that the route from their father’s bungalow to the car is less than 50m across a flat driveway, and that the route from the carpark to the coffee shop is similar, and both know that though their dad is getting frail he likes to walk when he’s up to it (Kittle, 2015a: 3019).

As stated in the cited work, in this scenario, Ann is “not asking whether Julian has the ability to walk up inclines of 10%, nor is she asking whether he can walk for 40 min straight or in heat of 40 ℃”, nor is she asking whether Julian can walk in “normal conditions” (whatever such might be) (Kittle, 2015a: 3019-3020). Moreover, Ann’s brother won’t mistakenly think she is: he will, rightly, take Ann to be asking whether Julian has the general ability to walk-very-short-distances-across-hard-flat-surfaces.

This point – that a general ability modal’s assessment conditions are not determined by a substantive notion of normal conditions – counts against any account of the general ability modals which appeals to a substantive notion of normal conditions (or similar) to do the work that I have suggested is sometimes done by the speaker’s intentions. It applies, for example, to Maier’s non-conditional, non-trying-based account of the general ability modals, which appeals to a set of assessment circumstances that are “similar to how things actually are” (Maier, 2015: 127). Like Mandelkern et al., Maier is explicit that what counts as “similar” circumstances must be determined by context (Maier, 2015: 128). But the question is: what notion of context is in play? If context includes the speaker’s intentions, the suggestion will work, but only because the notion of “normal” or “similar” conditions now does no substantive work and is simply a label for the set of circumstances fixed by the speaker’s intentions. If context excludes the speaker’s intentions, the solution won’t work because the general ability being ascribed may not pertain to circumstances which are similar to how things actually are.

It should be no surprise that appeal to a substantive notion of normal/similar conditions cannot do the required work since, as noted by Jansen (2007: 162–3), there is nothing problematic about asking what entities can do in distinctly^2008^* non-normal* or *abnormal* conditions: for example, when an army lieutenant asks whether the bridge *will be able to* hold the infantry company marching in step, the lieutenant is asking about what the bridge is capable of under distinctly abnormal conditions.

There is a parallel issue with respect to the ascription of dispositions. Many theorists have attempted to articulate what might constitute the “ordinary conditions” (Choi, ), “normal conditions” (Malzkorn, 2000), “ideal conditions” (Mumford, 1998: 87–92) for the assessment of any given disposition (e.g. fragility). I have argued elsewhere (Kittle, 2015b) that for dispositions too, the prejacent plus wider context (excluding speaker’s intentions) does not always or consistently determine a single set of assessment conditions, and that the solution to this problem is to allow speaker’s intentions to play some role in this (cf. Fisher, 2013). Again, this connection should be no surprise since ‘can’ and ‘is able to’ are often used to say what objects can do in virtue of their dispositions.

### Problem 3: Trying-Based Accounts Cannot be Extended to Cover All Types of Agent

Like Chisholm, Mandelkern et al. present a trying-based conditional analysis as a comprehensive analysis of the agentive modals. Given that particular/specific ability modals and general ability modals can be used to ascribe powers or abilities to non-minded agents which, on the face of it, cannot try, Mandelkern et al. have the job of explaining how to extend the account to cover these cases. In discussing this, they consider the following examples (attributed to Kieran Setiya, Maria Bittner and Martin Hackl respectively; my numbering):

(26) The flower can follow the sun.
(27) This elevator is able to carry three thousand pounds.
(28) This rock can tip the balance.

Examples (13), (18) and (19) pose the same problem:

- (13) Hydrolytic proteolytic enzymes are not able to digest fibrous proteins.
- (18) A warmer atmosphere is able to hold more moisture.
- (19) That asteroid can cause a mass extinction event on the earth.

Mandelkern et al. pursue a divide and conquer strategy for dealing with these cases. First, they suggest that “the notion of trying extends further than one might first imagine” such that “it is plausible … [to conceive] of the flower as *trying to follow the sun*” (Mandelkern et al., 2017: 336). Let’s grant Mandelkern et al. the idea that the notion of trying might be stretched to allow thinking of the flower as trying to follow the sun. Even so, this does not allow their analysis to predict the truth of (26) since it must also be the case that *following the sun* is a practically available action for the flower. And it is not plausible to think of the flower as *reasonably concluding in favour of following the sun given the goal of (following the sun? photosynthesising?)*, which is what their notion of practical availability requires. In response, Mandelkern et al. may point out (as discussed briefly above) that they explicitly state that the notion of practical availability is vague and sensitive to context, such that there may be cases where *practical availability* doesn’t require an instance of *reasonably concluding in favour of*.

But here we must question whether there is any notion of context which can do the required work: if the notion of practical availability is left so vague as to not always require the key notion in terms of which it was explained – i.e. reasonably concluding in favour of a course of action – then it seems that, rather than the analysis being to that degree contextual, the analysis is instead to that degree incomplete. We are given no account of how to contextually determine what counts as practically available, no rule or condition (context sensitive or otherwise) to apply once the context has been determined, and, moreover, we cannot employ any intuitive notion of practical availability because this notion, whatever it amounts to, is meant to be applicable to things which don’t perform actions.

On top of this, we should recall here the point made above in the treatment of the Chisholm-Lehrer objection, namely, that the “extra condition” in the analysans (i.e. the notion of practical availability) is, in the end, where the action is. Since *trying* just is *acting*, and since their analysis includes an instance of *trying* in the analysans, the problem identified here is not one which just affects a few fringe cases but one which affects the very core of the account: to the degree that their account leaves it unspecified what *being able to try* consists in, to that degree does their account leave it unspecified what *being able to act* consists in.

Mandelkern et al. propose to deal with examples (27) and (28) in a different manner, which they explain like so:

[(27)] can be glossed as saying, roughly, that there is some available action (… loading the elevator with three thousand pounds of freight and pressing ‘up’) such that in the closest world where the operator of the elevator tries to take that action, the elevator carries three thousand pounds. …

[(28)] can be glossed as saying, roughly, that there is some available action (… putting the rock on the balance) such that in the closest world where some relevant agent tries to take that action, the rock tips the balance (Mandelkern et al., 2017: 337).

The idea here is that the agent who does the trying need not be the subject of the ability ascription. The main problem with this suggestion is that it does not generalise, as both (18) and (19) illustrate. (19), for instance, plausibly expresses a claim about what the asteroid is able to do in virtue of its mass. And the case can easily be filled out such that there is no action available to any human which is such that, were it to be tried, the asteroid would cause a mass extinction event.

To this, Mandelkern et al. may respond that these are ascriptions of general ability modals and as such, the practically available actions being quantified over are not just those which might be practically available to those humans existing now, say, but those available to “normal agents in normal situations” across the full range of modal space. And perhaps some normal agents are such that, in their normal situations, they could try to do something which is such that, if it were tried, the asteroid would cause a mass extinction event on earth. But the response doesn’t work because there are versions of (26)-(28) and (20) which are particular/specific ability modals:

(26’) The flower can follow the sun now; I’ve just opened the curtain.

(27’) The repair is already complete, so this elevator is now able to carry three thousand pounds.

(28’) This rock can tip the balance right here, right now, even without anything else being added to the scales.

(19’) That asteroid is heading right for us, and it’s easily able to cause a mass extinction event on earth!

The lesson here is that the agentive modals can be used to ascribe a power or ability to an entity merely in virtue of the fact that the entity might act qua cause some change in another entity. If that’s right, then trying-based analyses will fail to generalise to all uses of the agentive modals.

## Summing Up

I have presented an in-depth assessment of conditional analyses of the agentive modals. The conclusion of this assessment is that philosophers were right to abandon this analysis, and that Mandelkern et al.’s recent presentation of the conditional analysis does not succeed in overcoming the problems identified. The root cause of the Chisholm-Lehrer objection was that trying-based compound analyses are committed to a constraint which means that there will inevitably be an instance of the analysandum in the analysans. This assessment suggests that an analysis of the agentive modals – at least if the desire is for a general analysis of all the agentive modals – should meet the following criteria: (i) it should accommodate the majority of a pre-theoretical intuitions across the range of uses to which the agentive modals may be put (a small but representative range of which were detailed in statements (1)-(22)); (ii) the analysis should not smuggle an instance of the analysandum into the analysans; (iii) the analysis should not tie possession of the ability to perform an action to a set of normal conditions. Criteria (i) and (ii) speak in favour of importing as few action-theoretic assumptions into the analysis as possible. And criterion (ii) also highlights the importance of being explicit about the concepts which are deployed in the analysans: if the notion of trying is appealed to in explicating what it is to be able to act, one should be clear about whether and in what sense trying is acting. Criterion (iii) suggests that, if the analysis appeals to context at some point, the nature of the notion of context invoked should be made plain. I have, in a companion piece to this article, expounded a relative modality-based analysis of the agentive modals which meets these criteria and addresses the standard objections to such accounts.^18^

## Footnotes

1 Austin (1956/1979: 229).

2 Adapted from van Inwagen (2017: 225).

3 Adapted from Mandelkern et al. (2017: 301).

4 The hyphenated notation comes from Bird (2000).

5 Throughout, I understand the modal’s prejacent to be the clause it embeds.

6 Ebner (2004: 275).

7 Fursule et al. (2009: 7).

8 Morgan (2015: 49).

9 LaFranchi (2020).

10 Martin and Shaw (2008: 113); the hope mentioned was realised in 2012.

11 UK Met Office Hadley Centre (2014).

12 This example is in the indicative mood and for that reason some (including myself) find it an unnatural sentence. In Kittle (n.d.) I present a positive, relative-modality-based account of the agentive-modals which explains this unnaturalness.

13 Adapted from Berlin (2002: 167).

14 Jennings (1965: 28).

15 Barr (2018).

16 Austin (1956/1979) discusses many such counterexamples.

17 Frankfurt called this the Principle of ***Alternate*** Possibilities. See my Kittle (2019: n. 2) for why ‘alternative’ should be preferred to ‘alternate’.

18 I would like to thank the work in progress reading group at the University of Leeds, as well as two anonymous reviewers, for comments on an earlier draft of this paper.
